# Efficacy of postoperative analgesia with intravenous paracetamol and mannitol injection, combined with thoracic paravertebral nerve block in post video-assisted thoracoscopic surgery pain: a prospective, randomized, double-blind controlled trial

**DOI:** 10.1186/s12871-023-02386-5

**Published:** 2024-01-04

**Authors:** Yin Zhou, Peng Yuan, Qi Xing, Wenjie Jin, Chonglong Shi

**Affiliations:** Department of Anesthesiology and Perioperative Medicine, The First Affiliated Hospital of Nanjing Medical University, 300 Guangzhou Road, Nanjing, 210029 China

**Keywords:** Paracetamol, Mannitol, Postoperative analgesia, Thoracic paravertebral block, Video-assisted thoracoscopic surgery

## Abstract

**Background:**

Although video-assisted thoracoscopic surgery (VATS) has advantages of reduced injury and faster healing, patients still endure moderate and severe postoperative pain. Paracetamol and mannitol injection, the first acetaminophen injection in China, has the advantages of convenient administration, rapid onset of action, and no first-pass effect. This aim of this study was to investigate the efficacy of postoperative analgesia with paracetamol and mannitol injection, combined with thoracic paravertebral nerve block (TPVB) in post VATS pain.

**Methods:**

This study was a single-center, prospective, randomized, double-blind controlled clinical trial. Patients scheduled for VATS were randomly divided into three groups, general anesthesia group (Group C), TPVB group (Group T) and TPVB + paracetamol and mannitol injection group (Group TP). In this study, the primary outcome was determined as visual analog scale (VAS) scores at rest and coughing, the secondary observation outcomes were the first time to use analgesic pump, the total consumption of oxycodone in the analgesic pump, number of effective and total analgesic pump compressions at first 48 h postoperatively, the perioperative consumption of sufentanil, time to extubation, hospital length of stay, urine volume, and the incidence of adverse events.

**Results:**

In a state of rest and cough, patients in the Group TP showed significantly lower VAS pain scores at 1, 12, 24, and 48 postoperative-hour compared with Group C and Group T. Intraoperative sufentanil and postoperative oxycodone consumption, the first time to press analgesic pump, the times of effective and total compressions of patient- controlled analgesia (PCA) were lower than those of the Group C and Group T. Interestingly, urine output was higher in Group TP. There were no differences between the three groups in terms of extubation time, length of hospital stay and adverse effects, indicating that intravenous paracetamol and mannitol injection is an effective and safe perioperative analgesia method.

**Conclusions:**

Paracetamol and mannitol injection, combined with TPVB may provide important beneficial effects on acute pain control and reduce the consumption of opioid in patients undergoing VATS.

**Trial registration:**

The trial was registered on Jun 19, 2023 in the Chinese Clinical Trial Registry (https://www.chictr.org.cn/showproj.html?proj=199315), registration number ChiCTR2300072623 (19/06/2023).

## Background

Currently, the thoracoscopic technique is widely employed in the field of thoracic surgery because of its reduced trauma, drainage volume, and blood loss, as well as faster recovery [1]. Although thoracoscopic surgery has less postoperative pain than thoracotomy, patients undergoing thoracoscopic surgery still endure moderate to severe postoperative pain [2]. This pain usually is caused by surgical incisions, intercostal nerve stretch, crush injury to the pleura and lung tissue. Notably, thoracic catheters often serve as a primary source of pain and discomfort for patients undergoing thoracic surgery, sometimes causing more pain than the incision itself due to intense pleural stimulation [3]. Inadequate pain control may increase the incidence of hypoxia, phlegm and pneumonia [4], which highlights the need for multimodal analgesia strategies to reduce the incidence of complications after thoracoscopic surgeries.

Effective postoperative analgesia is provided to patients with multimodal analgesia. Multimodal analgesia refers to the use of several different analgesic medications or techniques simultaneously, which can relieve postoperative pain, improve patients’ satisfaction, and shorten the hospital stay [5]. In this section, many analgesia methods are combined, and regional nerve block applications seem to become a cornerstone of multimodal analgesia with the rapid development of ultrasonic technology. Regional block applications can also be combined with a single injection in thoracoscopic surgery. In recent years, these applications have been frequently used under multimodal analgesia. Such as erector spinae plane block + thoracic paravertebral block, thoracic paravertebral block + intercostal block, deep serratus anterior block + superficial serratus anterior block, etc. With the continuous development of ultrasonic technology, ultrasound-guided thoracic paravertebral nerve block (TPVB) has been widely employed in thoracoscopic postoperative analgesia [6]. It has been confirmed that TPVB is an efficacious regional anesthetic method, providing sufficient analgesia to various surgical procedures, including pulmonary surgery [7, 8]. TPVB preserves respiratory and sympathetic function on the contralateral side and this may be related with reduced occurrences of hypotension, pulmonary complications, and urinary retention [9]. This regional nerve block, combined with patient-controlled intravenous analgesia, has become the mainstream of postoperative analgesia after thoracoscopic surgery.

With an understanding of the mechanisms of analgesia, we emphasize the combination of different classes of analgesics via different mechanisms to ensure a comfortable perioperative experience for patients. Opioids have been confirmed to be highly effective in controlling postoperative pain, yet accompanied by various adverse effects such as nausea, vomiting, respiratory depression [10]. Non-opioid options are frequently chosen to reduce opioid intake. Paracetamol and mannitol injection, the first intravenous acetaminophen injection in China, has the advantages of convenient administration, rapid onset of action, and no first-pass effect. Paracetamol exerts its analgesic effects via different pathways, including the inhibition of COX-2-dependent prostaglandin E2 synthesis, indirectly activating CB1 and TRPV1 receptors in the brain through its metabolites AM404 [11, 12]. Multiple studies have shown the positive effect of paracetamol on postoperative pain management in various procedures, including orthopedic surgery, radical mastectomy, cholecystectomy, cesarean section and abdominal hysterectomy [13, 14]. However, a previous study involving women receiving hysterectomy revealed that intravenous acetaminophen did not decrease narcotic use or postoperative pain compared with the group that received the placebo [15].

The purpose of this study was to investigate the postoperative analgesic effect of paracetamol and mannitol injection combined with TPVB for thoracoscopic analgesia. Patients who underwent thoracoscopic surgeries which included pulmonary lobectomy dissection, pulmonary wedge resection and segmentectomy were enrolled. We hypothesized that paracetamol and mannitol injection, combined with TPVB, would delay the time of the first use of the analgesic pump postoperatively, reduce perioperative sufentanil and postoperative remedial analgesic drug consumption, and decrease the postoperative visual analogue scale (VAS) score in patients undergoing thoracoscopic surgery.

## Materials and methods

This prospective study was approved by the Ethics Committee of the Jiangsu Province Hospital (2023-SR-217) and registered at the Chinese Clinical Trial Registry (ChiCTR2300072623) in accordance with the Helsinki Declaration. All patients signed an informed consent prior to participating in the research. A total of 120 patients undergoing video-assisted thoracoscopy surgery from June 2023 to November 2023 were enrolled.

### Inclusion Criteria

American Society of Anesthesiologists (ASA) I-II; age between 18 and 70 years old; body Mass Index (BMI) of 18 to 28 kg/m^2^; stable blood pressure control; normal liver and kidney function; coordinated with treatment.

### Exclusion Criteria

Chronic obstructive or restrictive lung diseases; severe central nervous system or cardio-cerebrovascular diseases; infections at block site; alcohol addiction; allergy to nonsteroidal anti-inflammatory drugs or local analgesics; using chronic analgesics; refusal to sign up for the trial.

### Termination Criteria

Transferring to thoracotomy; bilateral operation; perioperative bleeding exceeding 500 ml; postoperative transfer of the patient to the intensive care unit (ICU).

### Randomization and Blinding

In this randomized controlled study, eligible patients were randomly assigned to three groups according to the allocation sequence generated by computer program. The three equal groups were as follows: the general anesthesia group (Group C), the TPVB group (Group T), and the TPVB + paracetamol and mannitol injection group (Group TP), with 40 patients in each group. A bottle of intravenous paracetamol and mannitol injection is 500 mg (50 ml, H20223028, Jiangsu Hengrui Pharmaceuticals Co. Ltd). Before skin incision and 24 h after surgery, Group C and Group T received intravenous saline 50 ml, and Group TP received intravenous paracetamol and mannitol injection 50 ml. The intravenous infusion time in three groups was greater than 15 min. The anesthesiologists who were responsible for perioperative anesthesia management and the assessor who performed the follow-up was blinded to the group allocation. Collected data were recorded using a standardized study case report form and were analyzed by specialized staff.

### Operation of TPVB

All Patients were delivered to pre-anesthesia room and monitored by electrocardiography, heart rate (HR), blood oxygen saturation (SpO2) and non-invasive blood pressure (NIBP), then the peripheral venous access of the upper limb was opened by the nurses in charge. Patients in Group T and Group TP were placed in the supine position and ultrasound-guided TPVB was performed. TPVB was performed at T4 and T7 levels on the operative side via a SonoSite® S-Nerve portable ultrasound machine. After the syringle was gently withdrawn to confirm no air or blood, 15 ml of preconfigured local anesthetic (0.375% ropivacaine + 0.125 mg/ml dexamethasone) was injected into the patients in Group T and Group TP at each level. The block was confirmed successful by moving the probe up and down to check if local anesthetic distribution above pleura. All the blocks were performed by the same experienced anesthesiologists under the guidance of ultrasound in the pre-anesthesia room. After 30 min of observation without any discernible adverse reactions, the patient was transferred to the operating room.

### Anesthesia method

Before induction of anesthesia, all patients were deprived of food and water for eight hours, and the anesthesia monitors including electrocardiogram, HR, SpO2, respiration rate (RR), and invasive blood pressure (IBP). The patients then inhaled oxygen through a mask, and oxygen flow rate was set at 5L/min. For intravenous rapid anesthesia induction, ciprofol (0.4–0.6 mg/kg), midazolam (0.05 mg/kg), sufentanil citrate(0.5 μg/kg), penehyclidine hydrochloride (0.3 mg) and cisatracurium (0.2 mg/kg) were successively injected intravenously. After supplying oxygen through mask pressurization for 5 min, and double-lumen endotracheal tube intubation was performed under a visible laryngoscope. The endotracheal tube was located and identified under fiberbronchoscope, then the anesthetic ventilator was connected for mechanical ventilation. Before skin incision, the patients in Group C and Group T received intravenous saline 100 ml, and patients in Group TP received intravenous paracetamol and mannitol injection 500 mg + saline 50 ml. The parameters for single-lung ventilation were as follows: tidal volume of 6 mL/kg, respiratory rate of 14 times/min, inspiratory-to-expiratory ratio of 1:1.5, oxygen flow of 2L/min, and an end-tidal PCO2 of 35 to 45 mmHg. Anesthesia was maintained in three groups with remifentanil 0.06–0.2 µg/kg/min, propofol 4–6 mg/kg/h, cisatracurium 3 μg/kg/min, dexmedetomidine 0.2–0.4 μg/kg/h, and 1% sevoflurane inhalation. Hemodynamic variables were maintained within 20% of the baseline measurements. During the intraoperative period, when the MAP was < 80% of the base value, ephedrine 5 mg was administered intravenously; when the MAP was > 120% of the base value, urapidil 5 mg was administered intravenously; when the HR was < 80% of the base value, atropine 0.2 mg was injected intravenously; when the HR was > 80% of the base value, esmolol 5 mg was injected intravenously. After single lung ventilation, sufficient sputum aspiration and lung expansion were necessary. Approximately 30 min before the end of surgery, all patients received ondansetron 8 mg to prevent postoperative nausea and vomiting. After the operation, the patients were transferred to the PACU.

### Postoperative Management

When the patients regained consciousness and spontaneous ventilation, the tracheal tube was extracted, and patient-controlled intravenous analgesia was activated. The analgesic pump formula was 30 mg of oxycodone and 24 mg of ondansetron diluted to 150 ml with 0.9% normal saline. The analgesic pump was set to a background dose of 1 ml/h, a single patient-controlled additional dose of 6 ml, a locking time of 15 min and a maximum pump volume 30 ml/h. After extubation, the patient was closely monitored for stable vital signs and any signs of discomfort for 30 min before being transferred back to the ward.

### Observation index

Primary outcome: pain scores were assessed at the time points of 1, 6, 12, 24, and 48 h postoperatively. Postoperative pain was evaluated in a state of resting and coughing using visual analog scale (VAS) of 0–10, where 0 indicates no pain and 10 indicates the worst imaginable pain.

Secondary outcomes: the use of analgesic pump during the first 48 h postoperatively included the first time to press analgesic pump, the total consumption of oxycodone in the analgesic pump, the number of effective analgesic pump compressions, and the total compressions; the perioperative consumption of sufentanil; time to extubation (from the end of anesthesia to removal of the tracheal catheter); hospital length of stay, and urine volume. The incidence of adverse events included nausea and vomiting, dizziness, hypotension episode, liver function lesion and erythema within 48 h postoperatively.

### Statistical analysis

We conducted a preliminary study with 20 patients in each group and 60 patients in total. The pain scores of the three groups assessed at the time points of 1, 6, 12, and 24 h postoperatively were used to estimate the sample size. With a two-tailed α -error of 0.05 and a power of 0.8, the sample size was at least 34 patients per group under PASS15.0.5 software. Taking into account the 10% dropouts, the number of patients per group was increased to 40, a total of 120 patients. The data collected were statistically analyzed using the SPSS Statistics 21.0 (IBM®, NY, USA) software. The sample size was calculated with “Power and Sample Size.com,” an online power and sample size calculator. The measurement data were expressed as mean ± standard deviation or median (interquartile range), and the count data were expressed as frequencies (percentages). One-way analysis of variance was used to analyze the differences of the normally distributed continous variables, while the Kruskal–Wallis test was used to analyze the differences of non-normally distributed continous variables, then followed by Dunn’s multiple comparison tests. The Bonferroni method was used to correct the P-value in the pairwise comparison. *P* < 0.05 was regarded as statistically significant.

## Results

### General data

**Fig. 1:**
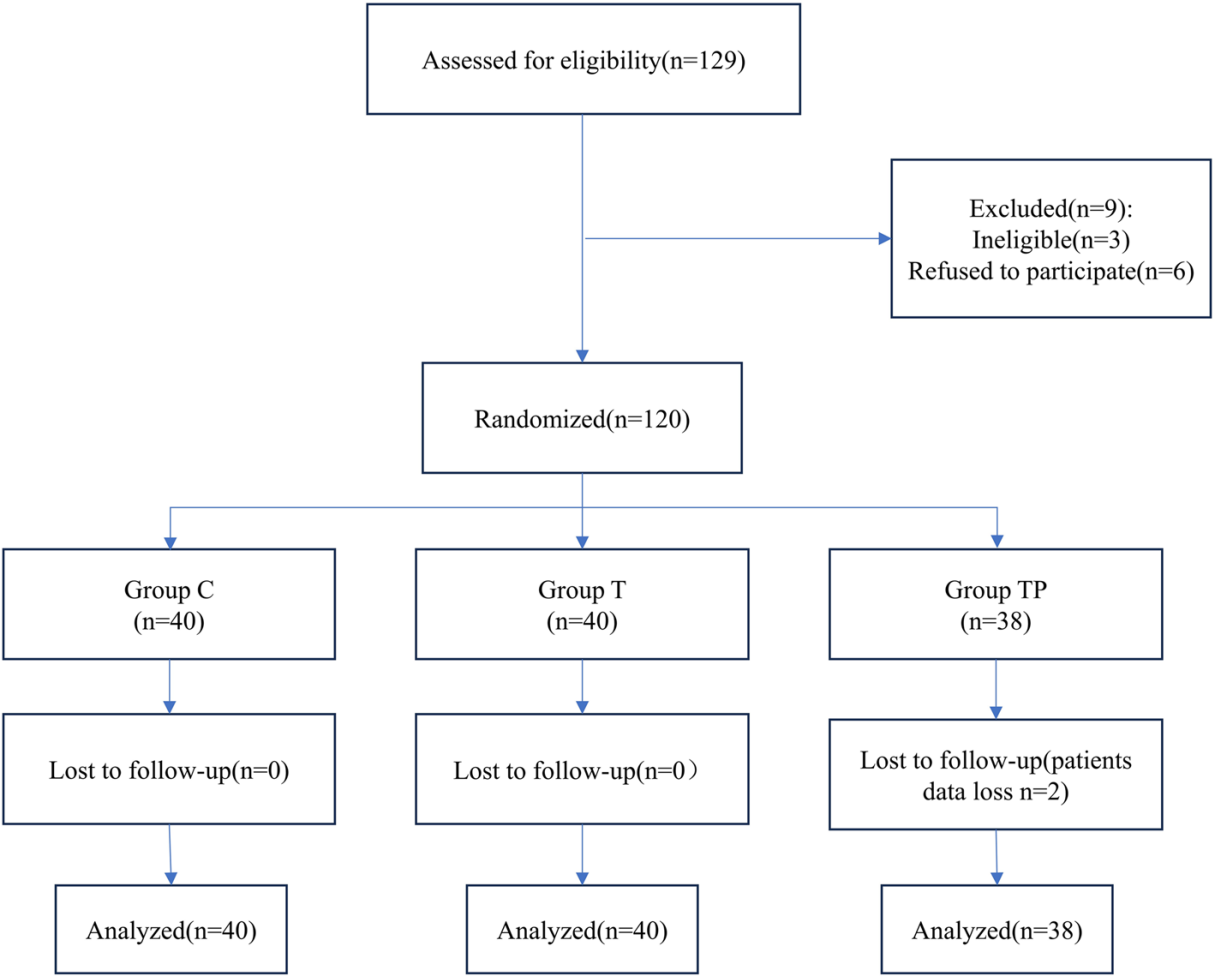
Flowcharts of the patients

In this study, a total of one hundred and twenty -nine patients were assessed for study eligibility between June and November 2023. Three patients did not meet the inclusion criteria and six patients refused to participate in the study. Finally, one hundred and twenty patients were selected. Three patients were excluded during the postoperative follow-up, resulting in 40 in the Group C, 40 in the Group T and 38 in the Group TP, as shown in Fig. 1. There was no significant difference in the demographic and operative data of the patients among the three groups (*p* > 0.05), as shown in Table 1. No complications directly relevant to TPVB and intravenous paracetamol and mannitol injection occurred.

**Table 1:** Demographic and surgical characteristics of the patients

Characteristic	Group C (n = 40)	Group T (n = 40)	Group TP (n = 38)	P-value
Age (years)	55.00 ± 11.37	54.28 ± 13.07	56.55 ± 9.72	0.673^a^
Sex male/female)	18/22	20/20	15/23	0.631^b^
Height(cm)	162.35 ± 6.46	163.75 ± 7.58	163.00 ± 7.55	0.686^a^
Weight (kg)	62.13 ± 9.90	62.80 ± 9.56	65.61 ± 10.60	0.273^a^
Body mass index (kg/m^2^)	23.49 ± 2.91	23.35 ± 2.65	24.65 ± 3.41	0.118^a^
ASA status (I/II/III) (n)	3/35/2	5/33/2	5/32/1	0.912^b^
Hypertension (n, %)	13(32.5)	14(35)	13(34.21)	1.000^b^
Diabetes mellitust (n, %)	3(7.5)	4(10)	3(7.89)	1.000^b^
Coronary heart disease (n,%)	4(10)	3(7.5)	3(7.89)	0.911^b^
Surgery duration (min)	76.78 ± 36.70	79.75 ± 33.39	88.26 ± 33.32	0.292^a^
Type of operation				
Wedge Resection	11(27.5)	10(25)	9(23.7)	0.991^b^
Segmenthectomy	10(25)	9(22.5)	9(23.7)	
Lobectomy	19(47.5)	21(52.5)	20(52.6)	
Infusion volume(ml)	1106.25 ± 338.58	1165 ± 276.01	1215.53 ± 280.30	0.277^a^

Values are mean ± SD or number of cases; ^a^ One-way analysis of variance;^b^ Pearson's chi-squared test; *ASA* American Society of Anesthesiologists, *BMI* Body mass index

### VAS scores assessments

The comparison among three groups demonstrated significant discrepancy in the VAS scores within each group at various time points after surgery. In a state of resting and coughing, patients in the Group T and Group TP showed significantly lower VAS pain scores at 1, 12, 24, and 48 postoperative-hour measurements compared with Group C. Pairwise comparisons indicated that the VAS scores in Group T were consistently higher over time compared with Group TP. Of note, there were no significant differences in the VAS scores under the rest and coughing condition at 48 h after surgery, as shown in Table 2.

**Table 2:** Pain scores at rest and coughing in the first 48 h

Outcomes	Group C (*n* = 40)	Group T (*n* = 40)	Group TP (*n* = 38)	*P*-value			
				C vs. T vs. TP	C vs. T	C vs. TP	T vs. TP
Valuation status Resting VAS							
1 h	2. 42 ± 0.93	1.85 ± 0.48	1.61 ± 0.55	< 0.001	0.003	< 0.001	0.115
6 h	3.25 ± 1.10	2.38 ± 0.49	2.16 ± 0.82	< 0.001	< 0.001	< 0.001	0.417
12 h	2.73 ± 0.72	2.20 ± 0.65	1.82 ± 0.61	< 0.001	0.002	< 0.001	0.034
24 h	2.42 ± 0.59	2.30 ± 0.52	1.89 ± 0.39	< 0.001	0.683	< 0.001	0.001
48 h	1.88 ± 0.56	1.88 ± 0.61	1.87 ± 0.41	0.998			
Cough VAS							
1 h	3.75 ± 1.08	2.93 ± 0.47	2.61 ± 0.55	< 0.001	< 0.001	< 0.001	0.022
6 h	4.65 ± 1.23	3.55 ± 0.50	3.16 ± 0.82	< 0.001	< 0.001	< 0.001	0.042
12 h	4.05 ± 0.78	3.33 ± 0.80	2.76 ± 0.71	< 0.001	< 0.001	< 0.001	0.005
24 h	3.80 ± 0.76	3.28 ± 0.51	2.61 ± 0.50	< 0.001	0.002	0.030	< 0.001
48 h	3.13 ± 0.65	3.03 ± 0.70	3.05 ± 0.57	0.772			

Values are mean ± SD, One-way analysis of variance; *VAS* Visual analog scale

### The time to first press PCA

Compared with Group C, the time to first press PCA pump was longer in Group T and Group TP. The pairwise comparison between Group T and Group TP manifested that the time to first press PCA pump was longer in groups Group TP, as shown in Table 3.

**Table 3:** The time to first press PCA, the oxycodone consumed postoperatively, and times of PCA

Variables	Group C (*n* = 40)	Group T (*n* = 40)	Group TP (*n* = 38)	*P*-value			
				C vs. T vs. TP	C vs. T	C vs. TP	T vs. TP
First press time(min)	45.5(10.5–212)	201(66.5–414)	563(371–830)	< 0.001^b^	0.001	< 0.001	0.002
Consumption of oxycodone in PCIA (mg)	23.36 ± 6.30	20.17 ± 5.13	15.90 ± 5.40	< 0.001^a^	0.013	< 0.001	0.001
Effective compressions of PCA (time)	15(9.5–18.5)	9(4–14.5)	5.5(3–8)	< 0.001^b^	0.005	< 0.001	0.031
Total compressions of PCA (time)	17(10.5–20.5)	10.5(5–16)	6(4–9)	< 0.001^b^	0.007	< 0.001	0.017

Values are mean ± SD or as median (interquartile range); ^a^One-way analysis of variance; ^b^Kruskal–Wallis test; *PCA* Patient-controlled analgesia, *PCIA* Patient-controlled intravenous analgesia

### Oxycodone Consumed Postoperatively

Compared with Group C, the consumption of oxycodone in PCIA was significantly lower in Group T and Group TP. Similarly, patients in Group TP received less oxycodone than those in Group T in the first 48 h after surgery, as shown in Table 3.

### Times of PCA

In comparison with Group C, the times of effective and total compressions of PCA were lower in Group T and Group TP within 48 h after surgery. Same as above, the times of effective compressions of PCA in Group T were higher than in Group TP. However, the total times of compressions of PCA showed no significant difference in Group T and Group TP, as shown in Table 3.

### Postoperative sufentanil consumption

Compared with Group C, the dose of sufentanil consumed during operation was lower in Group T and Group TP. However, the sufentanil consumption showed no significant difference in Group T and Group TP, as shown in Table 4.

**Table 4:** Sufentanil consumption, urine volume, time to extubation and length of hospital stay

Variables	Group C (*n* = 40)	Group T (*n* = 40)	Group TP (*n* = 38)	*P*-value			
				C vs. T vs. TP	C vs. T	C vs. TP	T vs. TP
Consumption of sufentanil (ug)	51.13 ± 5.94	45.88 ± 7.59	44.87 ± 5.63	< 0.001	0.001	< 0.001	1.000
Time to extubation (min)	30.13 ± 15.74	32.20 ± 12.88	28.05 ± 13.04	0.426			
Hospital length of stay (day)	6.15 ± 1.03	6.75 ± 1.45	6.55 ± 1.87	0.186			
Urine volume (ml)	224.50 ± 155.69	265.50 ± 199.77	407.11 ± 207.81	< 0.001	0.670	0.009	< 0.001

Values are mean ± SD; One-way analysis of variance

### Time to extubation and hospital stay

There was no significant difference in the length of hospital stay and the time of extubation among the three groups, as shown in Table 4.

### Urine volume during operation

In terms of urine volume, urine output of the Group TP was higher than that of Group C and Group T, as shown in Table 4.

### Incidence of postoperative adverse reactions

There were no significant differences in the incidence of nausea and vomiting, dizziness, hypotension episode, liver function lesions, and erythema among the three groups within 48 h after surgery, as shown in Table 5. In addition, no cases of local anesthetic poisoning, respiratory depression, or pulmonary infection occurred in any of the three groups.

**Table 5:** Incidence of postoperative adverse reactions

Adverse Event	Group C (*n* = 40)	Group T (*n* = 40)	Group TP (*n* = 38)	*P*-value			
				C vs. T vs. TP	C vs. T	C vs. TP	T vs. TP
Nausea and vomiting	8(20.0%)	5(12.5%)	6(15.8%)	0.673			
Dizziness	7(15.0%)	4(10.0%)	5(13.2%)	0.638			
Hypotension episode	1(2.5%)	4(10.0%)	5(13.2%)	0.207			
Liver function leision	0(0.0%)	0(0.0%)	1(2.6%)	0.321			
Erythema	1(2.5%)	0(0.0%)	2(5.3%)	0.316			

Values are number of cases; Pearson's chi-squared test

## Discussion

This study addressed the effect of paracetamol and mannitol injection (500 mg) intravenously administered before one-lung ventilation and 24 h after surgery, combined with TPVB, on post-thoracoscopic surgery pain. The main findings were that paracetamol and mannitol injection combined with TPVB can effectively decrease the 24-h postoperative VAS score and perioperative sufentanil consumption. It also led to a decrease in the consumption of oxycodone in PCIA, as well as a reduction in the times of effective and total compressions of PCA, while prolonging the time to the first postoperative analgesic administration in patients undergoing thoracoscopic surgery. In addition, there was no significant increase in the incidence of side effects related to paracetamol and mannitol injection.

Although the minimally thoracoscopic surgery markedly reduces pain degree, moderate to severe pain still persists after surgery, resulting in numerous complications such as postoperative pneumonia, atelectasis, and hypoxemia [16]. Patients undergoing thoracoscopic surgery tend to experience significantly acute and chronic postoperative pain, which can negatively affect respiratory function and life quality [17]. Previous researches have confirmed that TPVB, in combination with general anesthesia, can improve postoperative functional recovery in elderly patients undergoing thoracoscopic radical lung cancer surgery [18]. Despite the comfortable analgesic effect provided by TPVB, a certain amount of opioid adjuvant drugs is still needed to alleviate pain after surgery.

Multimodal analgesia protocol is necessary to relieve postoperative pain and reduce postoperative complications [19]. Multimodal analgesia involves different anesthesia techniques and a combination of various analgesics medications targeting multiple receptors within nociceptive and neuropathic pathways to prevent and manage pain [20]. In this study, patients undergoing thoracoscopic surgery were intravenously treated with paracetamol and mannitol injection during and after surgery, combined with TPVB, to reduce opioid consumption and alleviate acute pain.

To ensure the effectiveness and safety of postoperative analgesia, TPVB was performed by the same experienced anesthesiologists under the guidance of ultrasound. Numerous studies have confirmed that TPVB can reduce postoperative acute pain, mainly caused by the chest drain and opioid sparing after thoracic surgery [21, 22]. One previous study showed that general anesthesia combined with TPVB can reduce postoperative pulmonary complications by reducing postoperative pain in geriatric patients undergoing thoracic surgery [23]. In addition, the thoracic paravertebral block significantly shortened hospital stay after the intervention, which is critical for enhanced recovery program after surgery.

Postoperative pain scores were assessed by visual analogue scale (VAS). In our study, TPVB administered before general anesthesia reduced the intraoperative consumption of sufentanil, the postoperative VAS score in patients undergoing thoracoscopic surgery, prolonged the time to first postoperative remedial analgesia, reduced effective analgesic pump compressions, and postoperative oxycodone consumption compared with Group C. Our findings are consistent with previous studies that TPVB could achieve satisfactory postoperative analgesic effect. Of note, hypotension sometimes occurred in patients receiving TPVB, which might be related to the misplacement of paravertebral administered local anesthetics into the epidural space. However, we found TPVB did not shorten the hospital stay, which may be attributed to the fact that the incidence of postoperative overall insufficient analgesia tends to decrease after minimally invasive surgery. As is well known, the complications directly connected with TPVB were often observed, such as pneumothorax, puncturing of blood vessels, and local hemorrhage[24]. No complications directly associated with TPVB were reported in this study.

Patients experience severe pain due to the irritation of the thoracic duct within 24 h after thoracoscopic surgery, especially when patients cough or roll over [25]. Sometimes, TPVB alone is far from sufficient for postoperative analgesia, and multimode analgesia strategies should be administered to fulfill the purpose of enhanced recovery. As a frequently used antipyretic and analgesic drug, acetaminophen is commonly used in combination with other kinds of analgesics for multimodal analgesia [26]. Paracetamol and mannitol injection (50 ml, 500 mg), the first injection dosage form of acetaminophen in China, provides the advantages of no first pass hepatic metabolism and rapid onset, and is assumed to be safer than oral acetaminophen. The administration of 1000 mg of intravenous acetaminophen rapidly increases plasma concentration and results in higher peak concentrations than oral acetaminophen[27]. In our study, the patients received a recommended dosage of 500 mg of paracetamol and mannitol injection before one-lung ventilation, with two additional dosage administered 24 h after surgery. In a previous study of total knee arthroplasty, there was no clinical improvements or benefits in patients administered preemptive acetaminophen [14]. However, a retrospective study including 1416 patients revealed that the pretreatment of acetaminophen and gabapentin 30–60 min prior to operation reduced postoperative opioid requirements and pain [28].

In this study, the results showed the VAS scores of patients who were administered a combination of paracetamol and mannitol injection with TPVB were lower compared to those who received TPVB alone, especially at the coughing phase. There was no significant difference in the 1 h and 6 h after surgery, which can be attributable to the adequate analgesia provided by TPVB and not completely metabolized anesthetic. What’s more, the use of paracetamol and mannitol injection led to reduced intraoperative sufentanil consumption, postoperative oxycodone consumption, and the times of pressing the analgesic pump. These results indicate that the administration of paracetamol and mannitol injection combined with TPVB provides a more comfortable analgesic effect compared with TPVB alone. Interestingly, the urine volume of patients in Group TP was significantly higher than that in Group T, with no difference in fluid intake between Group TP and Group T. This may be caused by diuretic effect of mannitol and required us to pay attention to the intake and output of liquid during the surgery. Considering that acetaminophen is unstable and hydrolyzable in aqueous solution, mannitol is primarily used to regulate the osmolality of injectable fluids and has been shown to promote acetaminophen dissolution. Paracetamol and mannitol injection (50 ml, 500 mg) contains 1.925 g mannitol and the ratio of mannitol to acetaminophen content is 3.85. The concentration of mannitol in acetaminophen will not affect the patient's blood pressure and produce a slight diuretic effect. We also found that acetaminophen exerted the most significant analgesic effect within 12 to 24 h postoperatively. This may be because the acetaminophen is fully metabolized within six hours. Previous studies revealed acetaminophen hindered the enzymatic prostaglandin biosynthesis to release pain by inhibiting the cyclooxygenase 3 mainly [29]. The results of adverse events in this study indicate that the administration of paracetamol and mannitol injection did not increase the incidence of nausea and vomiting, which may be related to the fact that acetaminophen is a weak inhibitor of cyclooxygenase 1 and cyclooxygenase 2 [27]. Moreover, this dose did not lead to any liver function damage. The administration of paracetamol and mannitol injection before intraoperative single-lung ventilation exerted no effect on intraoperative hemodynamics and the duration of the operation. Additionally, paracetamol and mannitol injection with TPVB reduced use of sufentanil and the incidence of acute pain in the PACU.

This study also has certain limitations. Firstly, our sample included various thoracoscopic surgeries instead of focusing on a single radical lung cancer operation, which may have influenced the evaluation of postoperative analgesia. Secondly, the sample size of each group was relatively small and this study was conducted in single-center study, which may limit the generalizability of our results.

## Conclusions

In summary, our study shows that intravenous paracetamol and mannitol injection, combined with thoracic paravertebral block may provide important beneficial effects on acute pain control and reduce the consumption of opioid in patients undergoing thoracic surgery.

## Data Availability

No datasets were generated or analysed during the current study.

## Abbreviations

VATS: Video-assisted thoracoscopic surgery
TPVB: Thoracic paravertebral nerve block
PCA: Patient controlled analgesia
VAS: Visual analogue scale
ASA: American Society of Anesthesiologists
BMI: Body Mass Index
ICU: Intensive care unit
HR: Heart rate
SpO2: Pulse Oxygen Saturation
NIBP: Non-invasive blood pressure
IBP: Invasive blood pressure
